# Recent Advances in Photocatalysis–Microalgae Coupled Systems for Removing Recalcitrant Organic Pollutants from Water

**DOI:** 10.3390/nano16211335

**Published:** 2026-10-22

**Authors:** Lingtao Li, Xiaotong Zou, Miaosen Zhang, Yan Chen, Zhuo Zhang, Chi Zhang

**Affiliations:** 1 Faculty of Printing, Packaging Engineering and Digital Media Technology, Xi’an University of Technology, Xi’an 710054, China; 2 School of Materials Science and Engineering, Xi’an University of Technology, Xi’an 710054, China

**Keywords:** photocatalysis, microalgae, extracellular polymeric substance, pollutant degradation

## Abstract

Recalcitrant organic pollutants are characterized by stable molecular structures, high toxicity, and low biodegradability, making it difficult for a single treatment process to simultaneously achieve rapid transformation, extensive mineralization, and ecological safety. Microalgal treatment can support pollutant biotransformation, nutrient recovery, and carbon fixation, but its efficiency is often constrained by pollutant toxicity and limited bioavailability. Photocatalysis can rapidly break down stable molecular structures through reactive oxygen species, but may generate intermediates that resist further transformation. This review summarizes the working principles, system configurations, and performance-determining factors of photocatalysis–microalgae coupled systems for removing recalcitrant organic pollutants from water. Particular attention is given to photocatalytic activation, microalgal biotransformation, extracellular polymeric substance (EPS)-mediated interfacial regulation, and the effects of pollutant properties, material characteristics, microalgal physiological status, and reactor conditions. Recent advances show that the field is moving beyond pollutant removal efficiency as the sole endpoint and toward an integrated assessment of pollutant abatement, microalgal activity, and photocatalyst recovery. Mechanistic understanding has also progressed from empirical descriptions of synergy to systematic analyses of intermediate fate, interfacial electron transfer, and EPS regulation. Future studies should further elucidate pollutant transformation pathways and interactions at the nanomaterial–microalgae interface, and use these mechanistic insights to guide the coordinated design of photocatalytic materials, microalgal species, and reactor configurations, thereby improving treatment performance and long-term operational stability.

## 1. Introduction

Industrial production, agricultural activities, urbanization, and increasing pharmaceutical use continuously discharge wastewaters containing toxic and persistent pollutants, including phenols, antibiotics, endocrine-disrupting compounds, chlorophenols, dyes, and pesticides, into aquatic environments [1]. These compounds have stable structures, high toxicity, and limited natural attenuation; they can migrate and accumulate through food webs, posing long-term risks to aquatic ecosystems and public health [2]. Although osmosis, precipitation, ion exchange, membrane separation, electrochemical methods, and oxidation/reduction have been applied to wastewater treatment [3], their use in complex wastewaters is often constrained by high costs, incomplete removal, membrane fouling, and secondary waste generation [4]. Microalgal treatment can assimilate nitrogen and phosphorus, fix CO_2_, release oxygen, and contribute to pollutant transformation [5], but its performance against highly toxic, antimicrobial, or recalcitrant pollutants is limited by low bioavailability and toxic stress.

Under irradiation, photocatalysis generates reactive oxygen species (ROS) such as ·OH and ·O_2_^−^, which attack aromatic rings, conjugated structures, and stable chemical bonds [6]. The resulting ring opening, oxidation, dehalogenation, and chain scission convert complex pollutants into smaller intermediates [7]. However, photocatalysis alone often fails to achieve complete mineralization and may generate intermediates that remain toxic [8]. Photocatalysis–biological coupling has therefore attracted attention because rapid upstream activation can be followed by biological utilization and mineralization, compensating for the limitations of either process alone [9]. Microalgae are particularly attractive partners because they combine pollutant transformation, nutrient removal, oxygen production, carbon utilization, and stress-response capabilities [10].

Photocatalysis–microalgae systems have recently emerged as a focus of research. Photocatalytic materials not only catalyze reactions but also regulate light harvesting, electron transfer, interfacial contact, and the local reaction microenvironment [11]. Extracellular polymeric substances (EPS) secreted by microalgae can buffer oxidative stress, stabilize nanoparticle dispersions, adsorb pollutants and intermediates, and regulate interactions at the nano–bio interface [12]. Photocatalysis–microalgae coupling is therefore not a simple combination of two technologies, but a coordinated reaction network initiated by photocatalysis, sustained by microalgal metabolism, and buffered at the interface by EPS. This review focuses on coupled photocatalysis–microalgae systems for pollutant degradation. We discuss coupling modes, the fate of intermediates, EPS-mediated interfacial regulation, and the effects of reactor configuration on detoxification, mineralization, and system stability, and propose engineering design principles tailored to pollutant-specific bottlenecks.

## 2. Working Principles and Configurations

The defining feature of a photocatalysis–microalgae coupled system is not the simple serial connection of two treatment units, but the temporal and spatial coordination of pollutant transformation, intermediate consumption, oxidative pressure, and biological responses. Along material- and energy-transfer pathways, the system generally comprises four interconnected steps: pollutants migrate to and become enriched at the catalyst or extracellular interface; photocatalytic materials absorb photons and generate electron–hole pairs and ROS, converting stable organic structures into low-molecular-weight intermediates; microalgae and their associated microorganisms take up, assimilate, or further oxidize these intermediates while photosynthesis sustains oxygen and carbon exchange; and EPS together with reactor architecture regulates particle dispersion, interfacial contact, ROS exposure, and mass transfer. System performance should therefore be assessed in terms of pollutant transformation, product toxicity, degree of mineralization, microalgal activity, and material fate.

### 2.1. Fundamental Working Principles

Photocatalysis–microalgae coupling connects light-driven chemical transformation with biological conversion. Under irradiation, semiconductor photocatalysts generate electrons and holes. Electrons participate in oxygen reduction, while holes and derived reactive oxygen species initiate pollutant oxidation and structural cleavage. Microalgae process suitable transformation products through extracellular reactions, cellular uptake, and intracellular metabolism, contributing to further carbon conversion. The effectiveness of these coupled processes depends on the properties and biological fate of photocatalytic intermediates. The formation of biologically accessible products can facilitate subsequent conversion, whereas inhibitory or poorly biodegradable products may constrain biological treatment. Photocatalytic transformation and microalgal metabolism must therefore be coordinated to support intermediate consumption and sustain cellular activity. EPS contributes to this coordination by mediating material–cell interactions and local exposure to reactive species.

Synergism should be evaluated against the expected independent contributions of photocatalysis and microalgal treatment under comparable conditions. Photocatalysis-only, microalgae-only, and coupled treatments should be compared at matched initial pollutant concentrations, irradiation, pH, and component doses, with additional controls assessing adsorption and non-metabolic contributions. For simultaneous systems adequately described by independent parallel pseudo-first-order reactions, the sum of the background-corrected individual rate constants provides an additive benchmark. A coupled rate constant exceeding this benchmark beyond experimental uncertainty supports kinetic synergism, whereas agreement with it is consistent with additivity. Sequential systems require stage-specific comparisons at matched treatment time and energy input. Mineralization, intermediate profiles, and residual toxicity should complement parent-compound removal to establish whether kinetic enhancement improves treatment outcomes. Targeted perturbations of EPS availability, ROS generation, or electron transfer, together with measurements of intermediate turnover and microalgal activity, can further clarify the interactions responsible for the observed enhancement. These principles and evaluation criteria provide a basis for examining how different coupling configurations coordinate photocatalytic transformation and microalgal metabolism.

### 2.2. Configurations of Photocatalysis–Microalgae Coupled Systems

#### 2.2.1. Sequential Coupling

**Figure 1:**
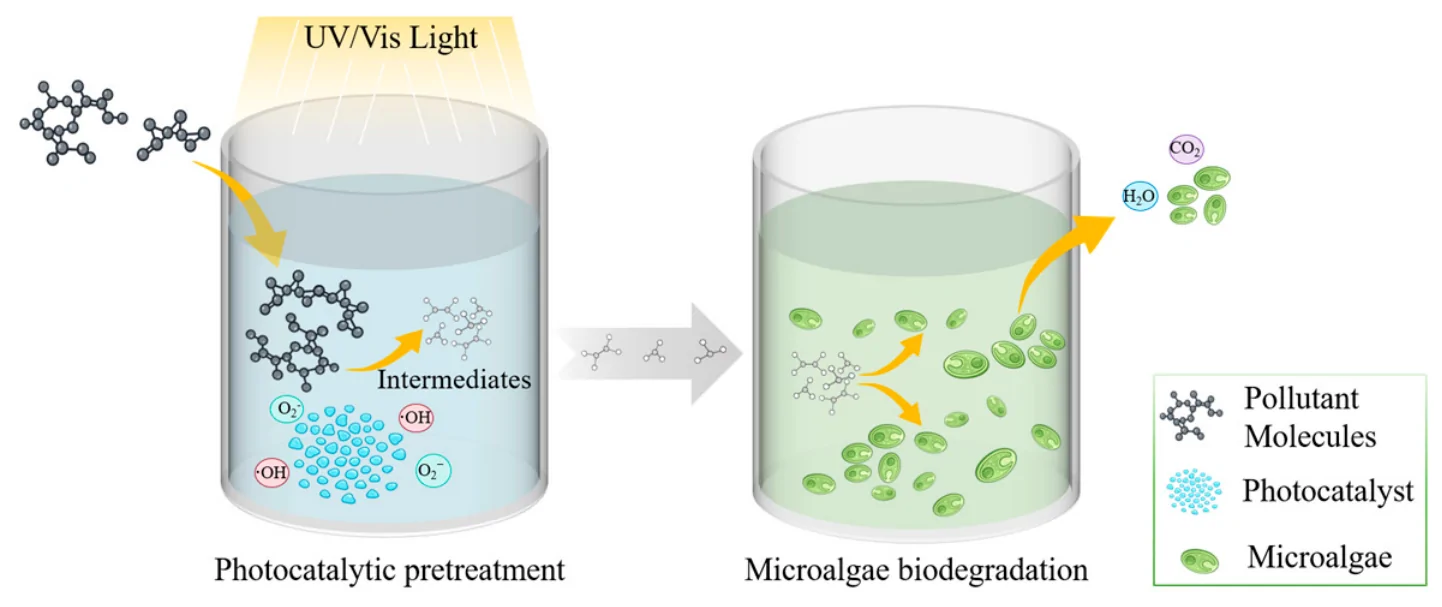
Schematic illustration of sequential photocatalysis followed by microalgal biodegradation for pollutant removal.

Sequential coupling generally comprises photocatalytic pretreatment followed by microalgal polishing (Figure 1). The first stage disrupts stable structures and reduces acute toxicity; the second takes up intermediates, removes nutrients, and promotes mineralization [13]. This arrangement allows pH, irradiation, and residence time to be controlled independently and prevents direct exposure of algal cells to strongly oxidative conditions. Its main limitation is that intermediates cannot be consumed as they are formed. Insufficient pretreatment leaves residual parent-compound toxicity, whereas excessive oxidation may increase energy consumption or generate products that are difficult to assimilate [14]. In a nitrobenzene system, p-nitrophenol accumulated during staged operation and inhibited subsequent biodegradation, showing that the pretreatment endpoint should be defined jointly by product toxicity and biodegradability rather than by pollutant removal alone [15].

Representative studies illustrate the potential of sequential coupling to coordinate pollutant transformation, microalgal treatment, and nutrient recovery. Photocatalytic pretreatment followed by microalgal treatment enhanced the mineralization of enrofloxacin [16]. A two-stage Nb_2_O_5_/UV-C pretreatment-microalgae process for Cr(VI)-containing wastewater illustrates the operational flexibility of staged treatment [17]. Studies on antibiotic-containing wastewater and florfenicol-containing aquaculture effluent further indicate that photochemical pretreatment followed by microalgal bioremediation can improve pollutant removal, toxicity mitigation, and nutrient recovery [18,19]. The value of functional separation lies in independently controlling upstream transformation and downstream biological treatment, but its effectiveness depends on the toxicity and biodegradability of the transferred intermediates. Related configurations extend beyond fully separated treatment units. Alternating photocatalytic membranes and algal biofilms provide a spatially integrated arrangement that can facilitate intermediate transfer between catalytic and biological zones [20]. A non-photocatalytic sequential anaerobic–aerobic algal–bacterial reactor also provides an engineering reference for combining upstream load reduction with downstream photobiological polishing [21]. Broader photocatalytic–biological studies offer additional guidance on separating treatment stages when oxidative conditions and biological operating requirements are incompatible [22,23]. The interstage transfer illustrated in Figure 1 highlights a key design question: how much photocatalytic pretreatment is required to improve biological treatability without accumulating inhibitory products? Future studies should relate the composition, toxicity, and biodegradability of the transferred intermediates to pretreatment duration and transfer or storage time, thereby defining conditions that support subsequent microalgal conversion. Where rapid intermediate consumption is essential, simultaneous or spatially integrated coupling may offer advantages over fully separated sequential operation.

#### 2.2.2. Simultaneous Coupling

**Figure 2:**
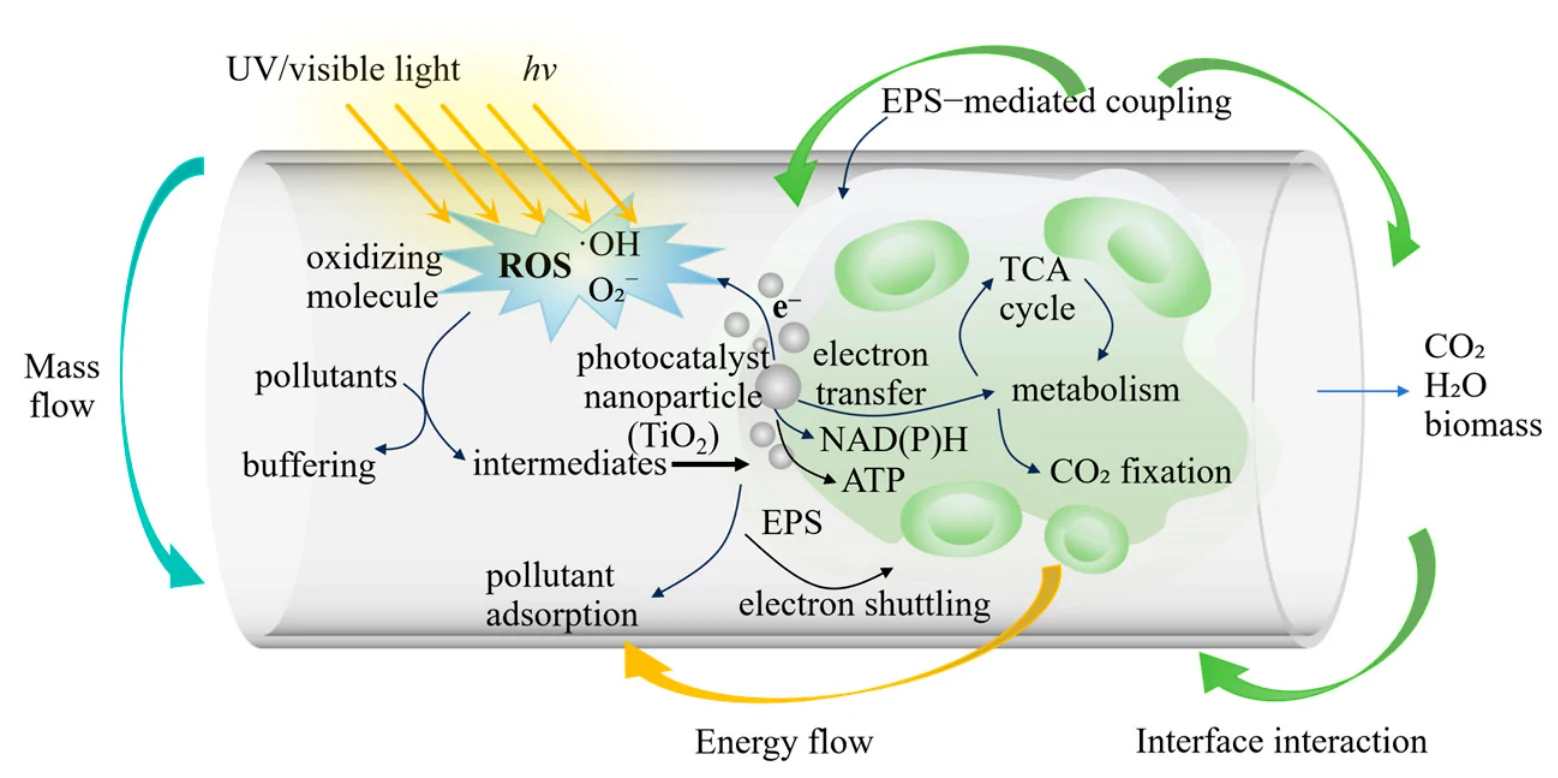
Schematic illustration of the synergistic mechanism by which photocatalysis and microalgae are coupled for pollutant degradation.

In contrast to sequential coupling, simultaneous coupling integrates photocatalytic conversion and biological metabolism within the same reaction space (Figure 2). Photoactive materials generate ROS and photogenerated electrons; the former attack aromatic rings, conjugated structures, and halogenated groups, while the latter may enter cellular electron-transfer chains through direct interfaces or mediators. The resulting low-molecular-weight products are rapidly utilized by microalgae, reducing interunit transfer and toxic accumulation [15,24,25]. The potential advantage of this mode lies in generating and consuming intermediates in situ, but spatial coexistence becomes true synergy only when oxidation, intermediate assimilation, and cellular defense occur on comparable timescales. EPS is the key interfacial layer that stabilizes simultaneous systems. Its polysaccharide, protein, and humic-like fractions can concentrate pollutants, regulate particle dispersion, participate in electron shuttling, and scavenge some ROS to mitigate cellular damage [8,26]. Stronger buffering is not always better: excessive coating can shield catalytic sites, whereas inadequate buffering exposes cells to oxidative pressure beyond their tolerance. EPS composition, thickness, and turnover should therefore be controlled together with material surface properties and ROS flux.

Simultaneous coupling requires a reactor arrangement that facilitates intermediate transfer while maintaining biological activity. ICPB reactors achieve close contact between photocatalysts and biofilms through shared carriers, allowing photocatalytically generated intermediates to undergo biological degradation and enhancing mineralization [25]. Layered photocatalytic biofilm reactors introduce spatial separation between the catalytic and biological zones while preserving intermediate exchange, thereby reducing direct oxidative stress on microorganisms [27]. The effectiveness of such arrangements also depends on establishing biomass on suitable supports. In attached microalgal cultivation, membrane properties influence cell adhesion and biomass accumulation, and XDLVO analysis has been used to explain these interactions [28]. Once the catalytic and biological components are positioned, light delivery and fluid circulation determine their access to photons and substrates. Tubular photobioreactors provide illuminated cultivation channels for controlled light delivery [29], whereas airlift reactors use gas-driven circulation to support mixing and CO_2_ supply [30]. These design features can be combined to connect photocatalytic conversion with microalgal metabolism. The coupled pathways illustrated in Figure 2 highlight the need to coordinate intermediate transfer and consumption with the maintenance of microalgal activity. Future studies should quantify how catalytic–biological spacing, support properties, illumination, and mixing jointly influence these processes, providing a basis for optimizing simultaneous coupling.

## 3. Functional Roles of Photocatalytic Materials

Photocatalytic materials influence coupled treatment through pollutant transformation and interactions with microalgae. Table 1 summarizes representative materials, algal species, treatment targets, and reported outcomes. The range of treatment targets requires material effectiveness to be evaluated according to the function being examined. The following sections connect these outcomes with material functions, energy conversion and electron transfer, interfacial regulation, and microalgal responses, providing a basis for identifying the processes that support effective coupling.

### 3.1. Photocatalytic Materials as Multifunctional Regulators

The studies in Table 1 can be considered in three groups: organic pollutant removal [33,34,39], nutrient and organic carbon removal [31,35,41,42], and metal removal [32,40,43]. These groups reflect different treatment functions and require different evidence to explain the material contribution. For example, the ZnO entries report contrasting hydrocarbon-removal outcomes for *Dunaliella tertiolecta* and Chlorella vulgaris at the same listed concentrations [34], suggesting that material effectiveness depends on the biological partner and pollutant loading. This highlights the need for matched comparisons that distinguish material-associated removal from biological contributions. For organic pollutants, transformation-product analysis and carbon balances would clarify whether disappearance leads to further conversion; for nutrient and metal removal, measurements of biological activity and pollutant partitioning would help resolve uptake, material binding, and chemical transformation. Such comparisons would connect the reported outcomes with the material functions responsible for them. In the *Chlorella ellipsoidea*@TiO_2_-Ag-AgCl nano-biohybrid, visible-light-driven photocatalytic activation promotes o-cresol degradation, while enhanced electron transfer and microalgal metabolic activity contribute to subsequent biotransformation [33]. Maintaining this biological contribution also requires sufficient energy and carbon supply. Carbon quantum dots can improve light utilization and photosynthetic electron transport, increasing carbon fixation and the accumulation of biomass and energy-storage compounds [44,45]. Carbon delivery can be further improved through material-assisted CO_2_ capture. Nanofiber membranes containing iron oxide nanoparticles adsorb CO_2_, extend its residence time, and promote dissolution, while providing a semifixed support for microalgal cultivation [46]. Together, these approaches connect material properties with the energy conversion and substrate supply that sustain microalgal metabolism. At the downstream stage, Fe_3_O_4_-based magnetic flocculation facilitates biomass harvesting and nanoparticle recovery, supporting the separation and reuse of process components [47,48]. The contribution of a material therefore depends on how its function complements the coupled process: photocatalytic activity initiates pollutant transformation, light and carbon regulation support biological conversion, and recoverability facilitates repeated operation. Combining these functions requires attention to both treatment performance and microalgal activity, together with the efficiency of material and biomass recovery.

### 3.2. Energy Conversion and Electron Transfer

Once the functions of photocatalytic materials have been defined, their regulation of light-energy input and electron flow must be considered. Energy-conversion efficiency depends primarily on material bandgap, charge-carrier lifetime, and the fate of interfacial electrons. g-C_3_N_4_ has a bandgap of approximately 2.7 eV and can drive initial pollutant oxidation under visible light [49]. Graphene oxide quantum dots can convert part of the ultraviolet spectrum into visible light at 400–700 nm; under indirect exposure, they increased microalgal biomass and bioenergy accumulation by 14.1% and 40.17%, respectively [50]. Nitrogen-doped carbon quantum dots promote pigment accumulation and nutrient removal, connecting microalgal photosynthetic responses with the nutrient-removal outcomes summarized in Table 1 [31], whereas self-assembled biohybrids reduce resistance to electron transfer, allowing photogenerated electrons to enter cellular metabolism more efficiently [51]. Material evaluation should therefore integrate spectral matching, electron utilization, ROS selectivity, and cellular energy status rather than rely on apparent degradation rates alone. Whether improved light utilization and electron transfer translate into treatment performance ultimately depends on the effective transport of electrons, ROS, and intermediates between photocatalytic materials and microalgae.

### 3.3. Engineering and Regulation of the Nano–Bio Interface

The nano–bio interface connects material photochemistry to microalgal metabolism and directly governs intermediate transport, interfacial electron exchange, and cellular tolerance. Polysaccharide, protein, and humic-like components of EPS regulate nanoparticle aggregation and distribution through adsorption, complexation, and surface coating [52]. In graphene oxide systems, proteinaceous and aromatic components affect material-cell binding [12], whereas under exposure to silver nanoparticles, amino and aromatic carboxyl groups participate in particle interactions and alter accumulation and toxicity [53]. These findings suggest that matching material surface chemistry with EPS composition is more important than merely minimizing the physical distance between materials and cells. Before nanoparticles contact cells, EPS can alter the aggregation state and surface properties of TiO_2_ [54,55]; it can also form an eco-corona on silver nanoparticles, reducing particle dissolution and bioavailability [56]. Algal EPS can further serve as a structural component in nano–bio composites, improving interfacial compatibility and promoting photocatalytic degradation [57]. Future systems could coordinate particle immobilization, substrate enrichment, electron exchange, and toxicity buffering through reversible material immobilization, layered arrangement of catalytic and microalgal units, and online characterization of interfacial processes. However, sustained synergy ultimately depends on the ability of microalgae to maintain physiological activity under continued exposure to ROS and nanoparticles.

### 3.4. Adaptive Responses of Microalgae to Photocatalytic Stress

As the biological endpoint of material photoelectrochemical and interfacial effects, microalgal acclimation determines whether the above material advantages yield stable and sustained pollutant removal. Microalgae do not passively endure photocatalytic stress; their physiological response varies with photocatalyst concentration, exposure mode, and interfacial conditions. For example, the effects of graphene oxide quantum dots (GOQDs) on *Chlorella pyrenoidosa* depend strongly on dose and exposure route. Direct exposure to 100 mg/L GOQDs increased biomass by 24.0%, whereas 1000 mg/L reduced biomass by 31% and caused oxidative stress and organelle damage. Indirect exposure retained the growth-promoting effect while reducing photooxidative injury [50]. Thus, the response is not simply inhibitory but reflects a balance between stress induction and adaptive regulation. Increased EPS secretion is a major protective mechanism: EPS adsorbs and encapsulates nanoparticles, buffers local ROS exposure, and regulates particle bioavailability, thereby reducing direct toxicity [52,53]. Under suitable interfacial conditions, EPS-mediated protection can preserve cellular integrity and may promote metabolic activation by improving electron utilization [51]. Microalgal responses also depend on the illumination regime. Material-assisted microalgal treatment and coupled systems have been operated with 12 h light/12 h dark or 16 h light/8 h dark cycles [31,33,35,36]. During darkness, light-driven ROS generation decreases, potentially relieving continued photooxidative exposure. Cellular recovery nevertheless depends on the preceding stress intensity, available metabolic energy, and the persistence of material-associated toxicity. Meanwhile, photosynthetic oxygen production stops while respiration continues, potentially lowering dissolved oxygen and constraining aerobic conversion when oxygen replenishment is insufficient. The contribution of dark intervals to system stability therefore depends on the balance between physiological recovery and treatment continuity. Comparisons of continuous and intermittent illumination at matched cumulative photon inputs, supported by measurements of PSII efficiency, intracellular ROS, dissolved oxygen, and pollutant conversion over repeated cycles, would help determine suitable light–dark operating conditions.

## 4. Applicability to Different Recalcitrant Organic Pollutants

The applicability of photocatalysis–microalgae coupling depends on the relationship between pollutant structure, catalytic transformation, and microalgal response. Table 2 summarizes representative chemical structures, experimental conditions, and treatment endpoints, providing a basis for interpreting the performance of selected coupled systems and related treatment approaches. The following sections examine pollutant-specific transformation characteristics and treatment limitations in the context of these conditions.

### 4.1. Phenolic and Chlorophenolic Compounds

Phenolic and chlorophenolic compounds occur in coking, petrochemical, papermaking, and pesticide wastewaters. Their treatment requires the conversion of aromatic structures together with control of the biological effects of the resulting intermediates. Photocatalysis–microalgae coupling connects initial chemical transformation with biological processing, making the balance between these steps central to the treatment of phenolic pollutants. Under the experimental conditions summarized in Table 2, the *Chlorella ellipsoidea*@TiO_2_-Ag-AgCl nano-biohybrid achieved complete removal of 100 mg L^−1^ o-cresol within 18 h. The average removal rate reached 5.56 mg L^−1^ h^−1^, compared with 0.857 mg L^−1^ h^−1^ for microalgae alone and 1.52 mg L^−1^ h^−1^ for the photocatalyst alone [33]. The bio-TiO_2_/Algae system containing *Tetradesmus obliquus* similarly achieved 98.95 ± 1.77% removal of 100 mg L^−1^ phenol within 19 h, compared with 40.88 ± 4.62% for microalgae alone under the same test conditions [58]. These results demonstrate the contribution of material-assisted transformation while retaining an active biological component.

Following rapid parent-compound removal, the subsequent fate of partially transformed aromatic products remains a key question. Increasing catalytic intensity can accelerate initial conversion, but the overall benefit depends on whether the resulting products undergo further biological treatment without suppressing microalgal activity. Intermediate profiles, mineralization, and toxicity should therefore be examined alongside removal kinetics. These measurements would help define operating conditions that match photocatalytic transformation to biological conversion and clarify how results obtained with phenol and o-cresol translate to other phenolic contaminants.

### 4.2. Antibiotics

Tetracycline, sulfonamide, and beta-lactam antibiotics exhibit strong antimicrobial activity and can inhibit microbial metabolism, limiting biological treatment alone. Photocatalysis–biological coupling first disrupts antimicrobial functional structures through photocatalysis, reducing biotoxicity and creating conditions for subsequent biological mineralization. For example, when Ag/TiO_2_ was coupled with activated sludge to treat 30 mg/L tetracycline hydrochloride, removal reached 85% within 8 h, markedly exceeding photocatalysis or biological treatment alone [8]. An intimately coupled Fe^3+^/g-C_3_N_4_ system removed 96.27% ± 5.27% of sulfamethoxazole and 86.57% ± 3.06% of COD [60], while readily degradable co-substrates improved antibiotic treatment in a related photocatalytic–biological system [61]. In the microalgal treatment example summarized in Table 2, direct UV pretreatment of florfenicol reduced toxicity and alleviated inhibition of nitrate assimilation in *Chlorella sorokiniana* [19]. These studies connect upstream antibiotic transformation with subsequent biological treatment, emphasizing the importance of relieving biological inhibition while promoting further degradation.

### 4.3. Dyes and Other Emerging Contaminants

Dyes and certain emerging organic contaminants are also important targets for photocatalysis–microalgae coupling and algae-supported photocatalytic treatment. Dyes often contain stable conjugated chromophores, so decolorization does not necessarily indicate complete degradation. Some emerging contaminants are persistent at low concentrations and may cause chronic toxicity; their evaluation must therefore consider the fate and detoxification of transformation products [62,63]. Cai et al. prepared a *Chlorella pyrenoidosa*/TiO_2_ composite by hydrothermal treatment, achieving 95.9% apparent Rhodamine B removal within 90 min under the visible-light irradiation conditions summarized in Table 2 [59]. Sorbent/photocatalytic granules incorporating non-living Chlorella vulgaris biomass and TiO_2_, developed by Blosi et al., completely transformed Rhodamine B within 1 h [64]. These studies illustrate the contribution of algal biomass to photocatalytic material design, while biological conversion in living microalgal systems additionally depends on cellular activity. For emerging contaminants such as bisphenol A and triclosan, a microalgal study examined removal processes together with estrogenic activity, demonstrating the value of assessing biological effects alongside chemical removal [65]. Performance for these pollutants should therefore be evaluated in terms of structural transformation, mineralization, and toxicity control rather than apparent removal alone.

Extending these applications to sustained operation requires conditions that support pollutant transformation while preserving microalgal activity. Light input, material dose, and residence time should be evaluated against intermediate composition, toxicity, and cellular responses, particularly under changing pollutant loads. Material stability also influences treatment performance: dissolution, aggregation, and accumulation can alter catalytic activity and biological exposure during repeated operation. Tracking material mass balances, recovery efficiency, and microalgal physiological responses would help determine whether initial treatment benefits can be maintained over successive cycles.

Wastewater composition introduces further constraints on practical applicability. Suspended solids, inorganic ions, and natural organic matter can modify light penetration, surface reactions, and transport, affecting the operating conditions established in laboratory tests. Continuous experiments with real wastewater should therefore evaluate treatment performance together with energy demand, material losses, and biomass-harvesting costs. Biomass utilization offers a further opportunity to improve process value, while multi-omics and in situ interface characterization can help identify the processes limiting sustained conversion. Linking these measurements with mineralization, residual toxicity, and operational stability would provide a stronger basis for assessing treatment feasibility and scale-up.

## 5. Conclusions

Coupling photocatalytic materials with microalgae can enable the synergistic transformation of recalcitrant organic pollutants. Photocatalysis first disrupts stable pollutant structures, producing more bioavailable and less toxic intermediates; microalgal metabolism then promotes further transformation, detoxification, and mineralization. EPS-mediated interfacial processes maintain biocompatibility by regulating particle dispersion, mass and electron transfer, and oxidative stress. Performance is therefore determined jointly by catalytic activity, microalgal tolerance, intermediate fate, and reactor configuration rather than by any single component. Evaluation should extend beyond apparent pollutant removal to include mineralization, product toxicity, microalgal activity, material recovery, and long-term operational stability. Because pollutants differ in structure, toxicity, and degradation pathways, system design must be tailored accordingly. Future studies should take a mechanism-driven approach, selecting visible-light-responsive and biosafe materials according to the major pollutant-specific bottleneck, regulating interactions between nanomaterials and microalgae, and optimizing reactor configuration and operating conditions. The synergy between photocatalysis and microalgae can be fully realized only when catalytic intensity, interfacial transport, biological tolerance, and reactor operation are appropriately matched.

## Figures and Tables

**Table 1:** Representative nanomaterial-assisted microalgal systems for pollutant removal.

Microalgal Species	Material	Pollutant	Removal Performance	Light Conditions	Ref.
*Chlorella sorokiniana*	N-CQDs	COD, TN, TP	17.6%, 5.3%, 7.9%	9000 lx; 12 h light/12 h dark	[31]
*Chlorella sorokiniana*	MnFe_2_O_4_	Cr^6+^, Co^2+^, Ni^2+^	100% (30 min, 5 mg/L), 27.3% (5 mg/L), 21.3% (10 mg/L)	-	[32]
*Chlorella ellipsoidea*	TiO_2_-Ag-AgCl	o-Cresol	Degradation rate increased by 549% relative to microalgae alone	Visible light; 4500 lx; 16 h light/8 h dark	[33]
	TiO_2_	o-Cresol	Degradation rate increased by 317% relative to microalgae alone		
*Dunaliella tertiolecta*	ZnO	Hydrocarbons	100% (0.05% *v*/*v*), 78.6 ± 1 (0.1% *v*/*v*), 42.5 ± 8 (0.5% *v*/*v*)	White fluorescent light; 110 μmol photons m^−2^s^−1^; continuous illumination for 72 h.	[34]
*Chlorella vulgaris*	ZnO	Hydrocarbons	100% (0.05% *v*/*v*), 97.5 ± 2% (0.1% *v*/*v*), 88.4 ± 6% (0.5% *v*/*v*)	Fluorescent light; 110 μmol photons m^−2^s^−1^; continuous illumination for 72 h	[34]
periphytic biofilm	TiO_2_	Nitrate	95%	150 W Xe lamp; 20 W m^−2^; 12 h light/12 h dark	[35]
periphytic biofilm	CdS	Nitrate	99.8%	Simulated solar irradiation; 20 W m^−2^; 12 h light/12 h dark.	[36]
*Chlorella vulgaris*	ZnO, Fe_2_O_3_	Ammonia; TP	decreased to near-zero levels after 100 days	16 h light/8 h dark	[37]
*Chlorella vulgaris*	Fe_2_O_3_	NH_4_^+^-N, PO_4_^3−^-P	92–99%, 36–68%	Outdoor cultivation under natural daylight	[38]
*Scenedesmus obliquus*	Al_2_O_3_	Sulfacetamide	18.8% (14 d, 50 mg/L NPs, 15 mg/L SA)	45–50 μmol photons m^−2^s^−1^; 16 h light/8 h dark	[39]
*Chlorella* sp.	Fe_3_O_4_	Cr(VI); Cu(II)	58.0% Cr(VI) and 73.4% Cu(II) removal after 6 h	-	[40]
*Scenedesmus obliquus*	CeO_2_	TP; TN	TP: 44.59–49.7%; TN: 72.1–85.6%	45–50 μmol photons m^−2^s^−1^; 16 h light/8 h dark	[41]
*Leptolyngbya* and *Nostoc*	CeO_2_	COD, TP, TN, Cu(II)	66.7%, 61.7%, 82.5%, 80.9%	2800 lx; 12 h light/12 h dark	[42]
*Synechocystis* sp. PCC6803	Fe_2_O_3_	Cr(VI), Cu(II), Pb(II), Cd(II)	88.37%, 78.89% 88.89%, 88.89%	-	[43]

**Table 2:** Chemical structures, experimental conditions, and treatment endpoints in representative photocatalysis–microalgae coupling and related treatment approaches.

Pollutant and Initial Concentration	Chemical Structure	Biological Component and Material	Light Source	Experimental Conditions	Treatment Time and Performance	Ref.
Phenol; 100 mg L^−1^	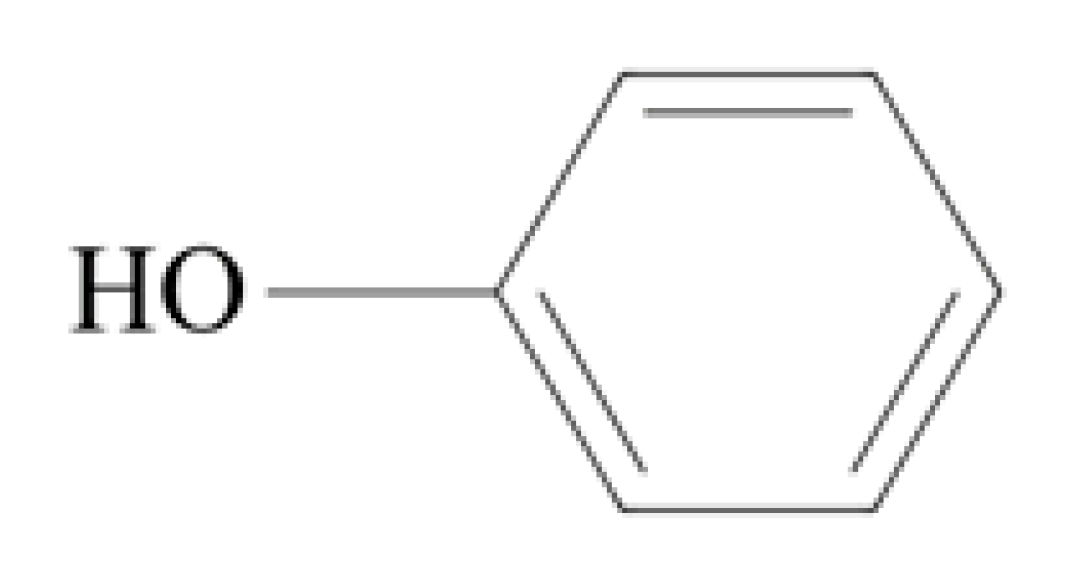	Living *Tetradesmus obliquus* coupled with bio-TiO_2_	4500 lx; 16 h light/8 h dark	BG11; pH 7.1; 30 °C; 60 mL in a 100 mL flask; 1% (*v*/*v*) inoculum (stock OD_680_ = 1.0).	Phenol removal (19 h): 98.95 ± 1.77% (living-algae hybrid), 40.88 ± 4.62% (microalgae alone), and 74.89 ± 1.12% (inactivated-algae hybrid).	[58]
o-Cresol; 100 mg L^−1^; additional tests at 200 and 300 mg L^−1^	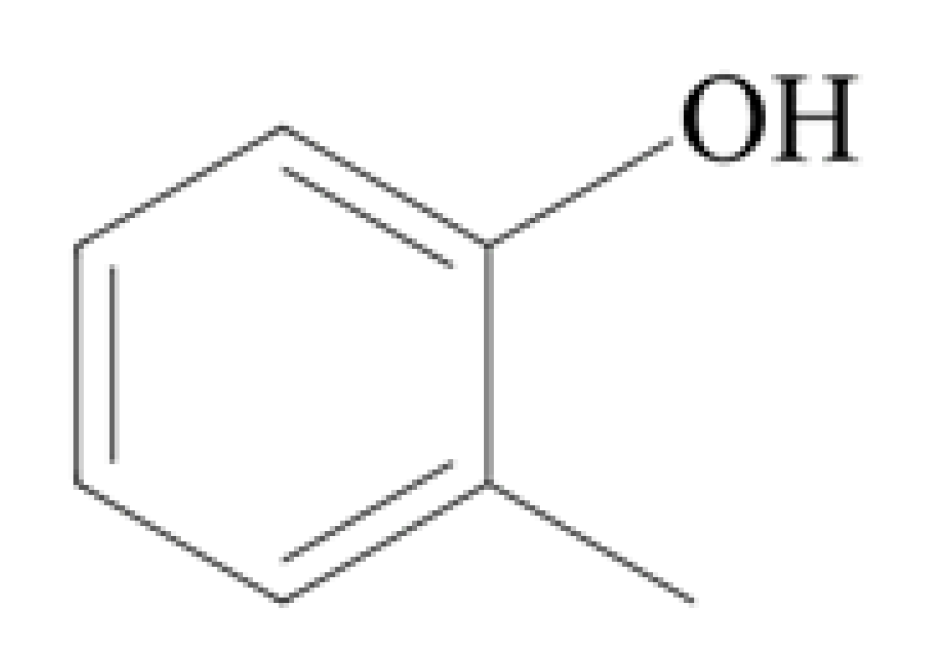	Living *Chlorella ellipsoidea*/TiO_2_–Ag–AgCl hybrid; prepared suspension diluted 1:120	4500 lx; 16 h light/8 h dark	BG11; pH 7.1; 30 °C; 100 mL flasks; algal control: 60 mL, OD_680_ = 1.0.	Complete removal of 100 mg L^−1^ o-cresol within 18 h; average removal rate of 5.56 mg L^−1^ h^−1^	[33]
Florfenicol; concentrations varied; 5 mg L^−1^ in the seawater kinetic test	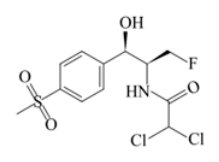	Living *Chlorella sorokiniana* following direct UV pretreatment; no photocatalyst	UV at 254 nm	seawater and aquaculture effluent	UV: >98% removal (60 min); rate constant: 0.1 min^−1^ (5 mg L^−1^, seawater).	[19]
Rhodamine B; 5 mg L^−1^	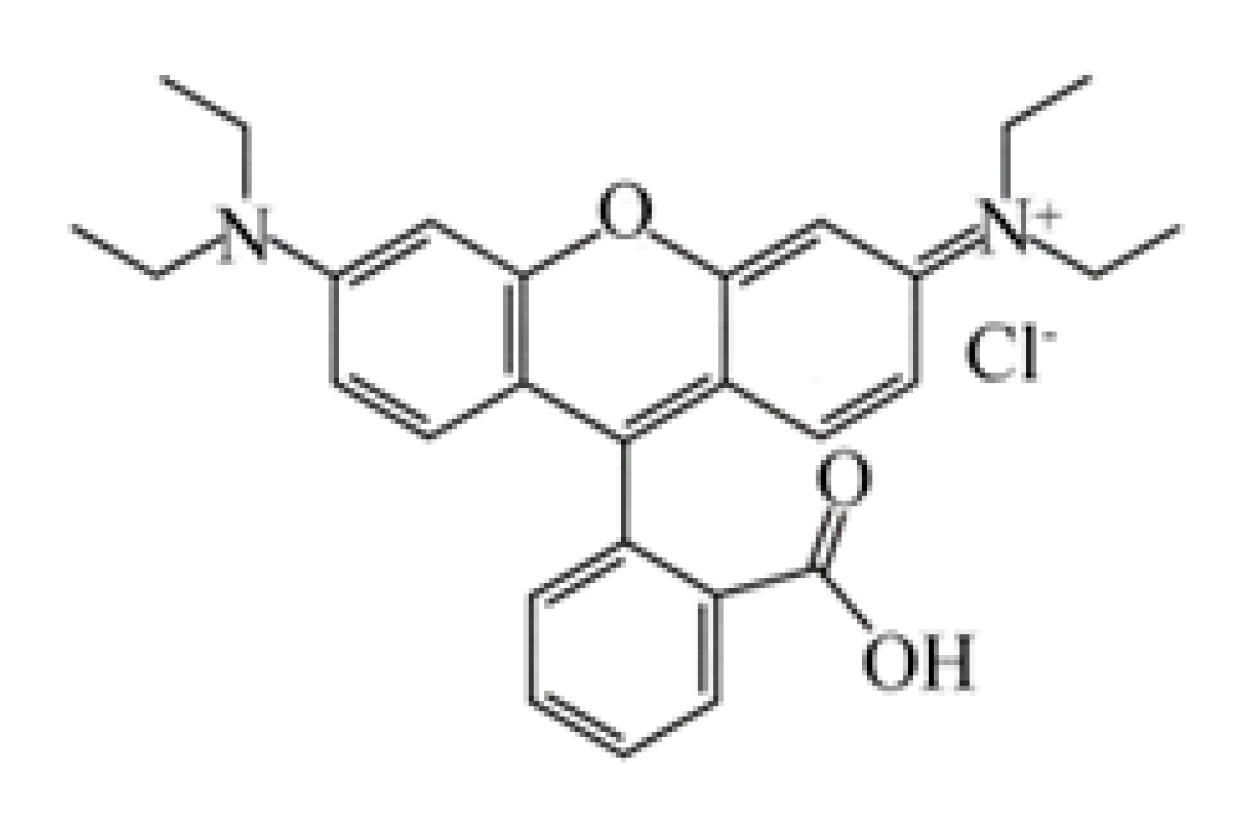	Hydrothermally prepared *Chlorella pyrenoidosa*/ TiO_2_ composite; algal biomass used as a support; composite loading of 1 g L^−1^	500 W Xe lamp with a 420 nm cutoff filter	30 min dark equilibration	95.9% apparent removal (90 min; absorbance at 553 nm).	[59]

## Data Availability

No new data were created or analyzed in this study. Data sharing is not applicable.
